# Management Strategies for POSEIDON Group 2

**DOI:** 10.3389/fendo.2020.00105

**Published:** 2020-02-27

**Authors:** Sesh Kamal Sunkara, G. A. Ramaraju, Mohan Shashikant Kamath

**Affiliations:** ^1^Division of Women's Health, Faculty of Life Sciences and Medicine, King's College London, London, United Kingdom; ^2^Center for Assisted Reproduction, Krishna IVF Clinic, Visakhapatnam, India; ^3^Department of Reproductive Medicine, Christian Medical College, Vellore, India

**Keywords:** POSEIDON classification, POSEIDON group 2, poor responder, suboptimal responder, IVF (ICSI)

## Abstract

Although individualization of ovarian stimulation aims at maximal efficacy and safety in assisted reproductive treatments, in its current form it is far from ideal in achieving the desired success in women with a low prognosis. This could be due a failure to identify such women who are likely to have a low prognosis with currently used prognostic characteristics. Introduction of the patient-oriented strategies encompassing individualized oocyte number (POSEIDON) concept reinforces recognizing such low prognosis groups and stratifying in accordance with important prognostic factors. The POSEIDON concept provides a practical approach to the management of these women and is a useful tool for both counseling and clinical management. In this commentary, we focus on likely management strategies for POSEIDON group 2 criteria.

## Introduction

Success following assisted reproductive treatments (ART) has improved significantly since the early years of *in vitro* fertilization (IVF) treatment. The notable contribution to this success is the introduction of ovarian stimulation into ART in the early 1980s (1, 2). Soon after the introduction of ovarian stimulation, it became apparent that women varied in their response to stimulation. To this end a low responder was first described as being associated with low serum oestradiol levels and requiring higher gonadotrophin stimulation doses (3). Since then, there had been varied descriptions and terminologies such as poor ovarian response (POR), low response, inadequate response, suboptimal response with numerous definitions, several criteria and different thresholds.

A review in 2000 enlisted around 28 criteria used for the definition of POR (4) and a latter review nearly 10 years later reinforced the issue of lacking uniform criteria for defining POR with 41 definitions being used in 47 RCTs that had since been published on the topic (5). Discrepancies in the definition lead to clinical heterogeneity among studies on POR leading to inconsistent and inconclusive findings (6). This lead to researchers and clinicians calling for a unified definition of POR leading to the publication of the ESHRE consensus, Bologna criteria definition of POR (7).

However, there has been skepticism whether the ESHRE consensus, the Bologna criteria for defining POR is fit for purpose and whether the new consensus definition mitigated clinical heterogeneity. The ESHRE consensus definition of POR considers proven poor responders based on previous cycle performances and predicted poor responders as one category, does not consider suboptimal response, does not factor in female age and the oocyte competence in terms of embryos aneuploidy rate (8). Additionally, it comprises of several subpopulations with varied baseline characteristics (9). Furthermore, the Bologna criteria encompasses a very poor prognosis group that is associated with very low live birth rates (10, 11) raising the interrogation if any interventions could enhance clinical outcomes for these women with very poor prognosis (12, 13).

## Concept of Individualized Ovarian Stimulation

The main objective of individualization of ovarian stimulation (OS) is to offer women the best treatment tailored to her own unique characteristics, thus maximizing the chances of pregnancy and eliminating the iatrogenic and avoidable risks resulting from ovarian stimulation (14). It currently entails categorizing women based on their predicted response in order to individualize OS regimens. Women can be identified as having an expected poor response, normal response or a high response based on ovarian reserve tests (ORTs). Among the various ORTs including basal FSH, basal oestradiol, inhibin B, antral follicle count (AFC), and anti-mullerian hormone (AMH), AFC, and AMH have the highest accuracy for the prediction of either a poor or a high response following ovarian stimulation (15–17). However, whether the categorization into the three broad categories of poor, normal and high response is sufficient to categorize all women in an ART programme has been questioned with evidence-based suggestions to refine the categorization by recognizing the suboptimal responder (18). Suboptimal response is the group between poor response with ≤3 oocytes and normal response with 10–15 oocytes. These women with 4–9 oocytes have a better prognosis over poor responders but have a lower prognosis compared to normal responders. Given that poor responders have a very low prognosis with most interventions being futile, it would be justifiable to focus research and interventions toward other low prognosis groups such as the suboptimal responder. This further lead to the notion of “patient oriented strategies encompassing individualized oocyte number”—POSEIDON concept. The recent publication in Frontiers in Endocrinilogy by Conforti and colleagues hughlights the need for intervention studies to test the POSEIDON concept, particularly for groups 1 and 2 where benefit is more likely in the context of a good ovarian reserve (19). This paper discusses interventions in the context of POSEIDON group 2 women that would merit further research.

## Poseidon Concept: the Why, the What and the How

A systematic review and meta-analysis on predictive factors in IVF evaluated nine common predictors and found the following factors of female age, duration of infertility, basal follicle stimulating hormone (FSH) levels, the number of retrieved oocytes, and embryo quality to be associated with the chances of pregnancy (20). Older female age, longer duration of infertility, higher basal FSH levels were negative predictors whereas higher number of oocytes and good embryo quality were positive predictors. There has been consistent evidence of a strong association between number of oocytes retrieved and live birth reinforcing that the number of oocytes is an important prognostic variable for IVF success (21–24). It is therefore paramount that the OS regimens optimize number of oocytes retrieved to maximize success.

Younger women have a favorable prognosis in achieving a live birth compared to older women. An important reason for this is the increase in aneuploid embryos and consequent decrease in euploid embryos with increasing female age. Whereas, female age influences the embryo euploidy rate, euploidy rate remains stable in relation to the embryo cohort sizes, thereby resulting in more euploid embryos with higher number of embryos (25). It therefore becomes apparent that female age, ovarian reserve, ovarian response to stimulation and number of oocytes retrieved are overriding factors determining the success of ART. Ovarian stimulation regimen should therefore aim to enhance ovarian response to stimulation, particularly for the low prognosis group of patients extending to the suboptimal responder.

The POSEIDON stratification is aimed at clinical management by considering the most important prognostic factors and stratifying women accordingly. Group 1: women aged <35 years with adequate ovarian reserve (AFC ≥ 5, AMH ≥ 1.2 ng/ ml) with an unexpected poor response (<4 oocytes) or a suboptimal response (4–9 oocytes); Group 2: women aged ≥ 35 years with adequate ovarian reserve (AFC ≥ 5, AMH ≥ 1.2 ng/ ml) with an unexpected poor response (<4 oocytes) or a suboptimal response (4–9 oocytes); Group 3: women aged <35 years with poor ovarian reserve (AFC < 5, AMH < 1.2 ng/ ml); Group 4: women aged ≥ 35 years with poor ovarian reserve (AFC < 5, AMH < 1.2 ng/ ml) (26).

## Mangament of Poseidon Group 2 Women

The aim of defining the POSEIDON groups is to individualize therapeutic approaches by fine tuning OS in terms of the right pituitary suppression regimen, the ideal gonadotrophin selection, along with dosage and optimize ovarian response and number of oocytes to obtain a euploid embryo with the highest implantation potential for transfer. POSEIDON classification reinforces the avoidance of iatrogenic suboptimal response underpinning likely genetic variants such as FSH receptor polymorphism (27), variant luteinising hormone–β (V LH–β) (28) that might benefit from gonadotrophins with different pharmacokinetic profiles and yielding a higher number and competent oocytes for a given dosage (29, 30). The broad stratification based on a female age cutoff, taking cognizance of the declining prognosis in women beyond age of 35 years, is likely to be helpful in clinical decision making for the vast majority of women undergoing IVF below age of 40 years (31).

The long gonadotrophin releasing hormone (GnRH) agonist regimen is associated with a significantly higher oocyte yield over the short GnRH agonist regimen, and as such should be the preferred downregulation regimen with the use of GnRH agonists (32). Given the concurring evidence of comparable efficacy with the use of the long GnRH agonist and GnRH antagonist regimens for both general population and poor responder women undergoing IVF (33), either regimens could be recommended for POSEIDON group 2 women by extrapolating current evidence. *As the aim is to improve egg numbers, these women may benefit from a higher gonadotrophin dose over the standard 150 IU – 225 IU daily for OS* (34). There is the discussion that women in this group have a specific genotype profile accounting for the variability in ovarian response to stimulation that is unexpected based on routine ovarian reserve testing. *There has been evidence on the relevance of genetic variants of gonadotropins and their receptors in ovarian stimulation and benefits of increasing FSH dose or adding recombinant LH for women with a hypo response to recombinant FSH* (35) *depending upon presence of FSH or LH receptor polymorphisms, respectively* (36). *Whether FSH or LH receptor polymorphism screening should be offered to all women with adequate ovarian reserve prior to their first IVF treatment depends on prevalence of such polymorphism in this select IVF population and its impact. Further, whether these women are likely to benefit from gonadotrophins with different pharmacokinetic profile such as additional LH activity to FSH needs evaluation with further research into this area*.

Dual stimulation is a novel strategy in which double stimulation (“DuoStim”) is attempted in the same menstrual cycle (36). Earlier, it was proposed that only one wave of follicular recruitment takes place in an ovarian cycle. It has hence been shown that two and three cohorts of antral follicles are recruited during a menstrual cycle (37–39). Double stimulation has been proposed as one of the treatment statergy for management of POSEIDON group 2 which consists of women ≥35 years with an unexpected POR or suboptimal response. Since aneuploidy rates are higher in this group compared to women <35 years, higher oocyte yield is needed to achieve a single euploid embryo. Double stimulation strategies can help in maximizing oocyte yield in a single ovarian cycle. An earlier study has compared the oocyte yield and euploid blastocyst rates following FPS and LPS (40). The study reported no significant difference in retrieved cumulus oocyte complex (5.1 ± 3.4 vs. 5.7 ± 3.3) or euploid blastocyst rates (46.9 vs. 44.8%). A recent case control included 188 women with poor prognosis who under double stimulation (41). The authors reported fewer oocytes collection (3.6 ± 2.1 vs. 4.3 ± 2.8; *P* < 0.01) and euploid blastocysts (0.5 ± 0.8 vs. 0.7 ± 1.0; *P* = 0.02) after FPS compared to LPS. A systematic review which included eight studies and 338 women, reported no compromised in quality or quantity of oocytes retrieved following LPS compared to FPS (42). A “freeze all strategy” is mandatory for double stimulation. It is suggested that double stimulation may reduce the cycle drop out rates in these women with poor or suboptimal response and shorten time to pregnancy (42). Currently, there is very limited data is available on obstetrical and neonatal outcomes following double stimulation.

**Figure 1:**
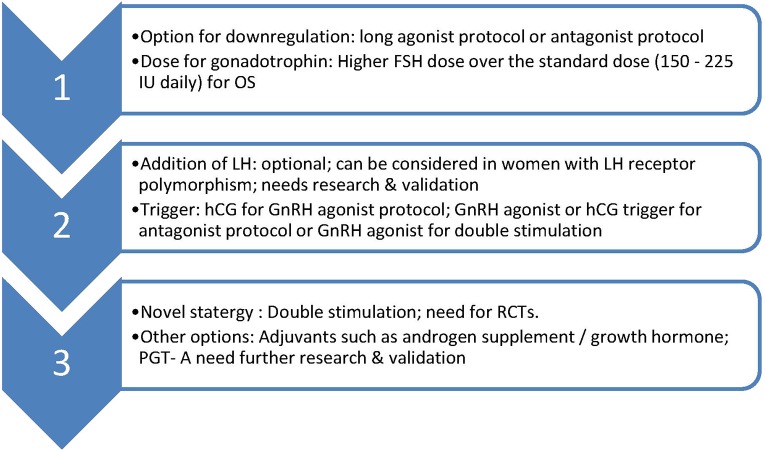
Suggested management of POSEIDON group 2. OS, ovarian stimulation; LH, luteinising hormone; hCG, human chorionic gonadotrophin; GnRH, Gonadotrophin releasing hormone; PGT-A, pre implantation genetic testing-aneuploidy; RCT, randomized controlled trial.

Double stimulation protocol needs validation in POSEIDON group 2 population along with cost-effectiveness and safety data. *Overall, such group of women in POSEIDON group 2 might benefit recognition and whether they could benefit from novel strategies or inteventions such as adjuvant androgen therapies, addition of growth hormone, preimplantation genetic testing for aneuploidy (PGT-A) warrants further research*. Management options for POSEIDON group 2 women is summarized in Figure 1.

## Author Contributions

SS drafted the manuscript with contribution from GR and MK. All authors approved final version.

### Conflict of Interest

The authors declare that the research was conducted in the absence of any commercial or financial relationships that could be construed as a potential conflict of interest.
